# Genomic and Phenotypic Evolution of Tigecycline-Resistant Acinetobacter baumannii in Critically Ill Patients

**DOI:** 10.1128/spectrum.01593-21

**Published:** 2022-01-19

**Authors:** Jiangang Zhang, Jinru Xie, Henan Li, Zhiren Wang, Yuyao Yin, Shuyi Wang, Hongbin Chen, Qi Wang, Hui Wang

**Affiliations:** a Department of Clinical Laboratory, Peking University People’s Hospital, Beijing, China; University of Pittsburgh School of Medicine

**Keywords:** *Acinetobacter baumannii*, tigecycline resistance, within-host evolution, virulence

## Abstract

Acinetobacter baumannii is an important opportunistic pathogen of nosocomial infections. A. baumannii presently exhibits increasing antibiotic resistance, which poses great challenges to public health. The occurrence of tigecycline-resistant A. baumannii is related to tigecycline treatment and the within-host evolution of bacteria. We analyzed isogenic A. baumannii isolates from two critically ill patients who underwent tigecycline treatment. Whole-genome sequencing and comparative analyses were performed to determine the characteristics of genomic evolution. We conducted phenotypic studies, including *in vitro* antibiotic sensitivity tests, biofilm formation tests, growth curve determination, serum bactericidal determination, and Galleria mellonella lethality assays. *In vivo* emergent tigecycline resistance was observed after tigecycline treatment. After the withdrawal of tigecycline pressure, tigecycline-resistant isolates were not isolated from one patient. Four tigecycline-resistant isolates exhibited lower growth rates. The biofilm formation and virulence characteristics of tigecycline-resistant isolates were reasonably different between the two patients. A special phenotype appeared after tigecycline treatment in both patients, accompanied by reduced serum tolerance, enhanced biofilm formation ability, and reduced virulence of Galleria mellonella. Most of the genomic variation occurred after the tigecycline treatment, primarily involving transcription-, signal transduction-, translation-, ribosomal biogenesis-, and cell wall biogenesis-related genes. We determined that the genomic variations in *baeR*, *wzc*, *aroQ*, *rluC*, and *adeS* and acquisition of IS*Aba1* were associated with tigecycline resistance *in vivo*. Capsular polysaccharide-related genes, *wzc*, and *itrA2*, and *aroQ*, were the key genes related to the virulence evolution of A. baumannii within the host.

**IMPORTANCE** Multidrug-resistant Acinetobacter baumannii poses a huge challenge to clinical treatment, and tigecycline is considered a last-line drug for the treatment of multidrug-resistant A. baumannii. However, the mechanism of tigecycline resistance *in vivo* has not been elucidated. This study analyzed the genomic and phenotypic evolution of tigecycline-resistant A. baumannii in two critically ill patients. In this study, after treatment with tigecycline, tigecycline-resistant A. baumannii emerged with higher fitness costs. After the withdrawal of tigecycline pressure, tigecycline-resistant isolates were not isolated from one patient. The *in vivo* and *in vitro* virulence of the isolates exhibited diametrically opposite results in the two patients. Genomic variations in *baeR*, *wzc*, *aroQ*, *rluC*, and *adeS* and acquisition of IS*Aba1* were associated with tigecycline resistance *in vivo*. The capsular polysaccharide-related genes, *wzc*, *itrA2*, and *aroQ*, were the key genes related to the virulence of A. baumannii in hosts. Our research provides a theoretical basis for elucidating the mechanism of tigecycline resistance and presents new clues for future surveillance and treatment of multidrug-resistant A. baumannii.

## INTRODUCTION

Acinetobacter baumannii is an opportunistic pathogen widely distributed in nature and in hospital environments, capable of colonizing the human skin, oral cavity, respiratory tract, and gastrointestinal tract. It is one of the most important pathogens in nosocomial infections, mainly causing pneumonia, meningitis, urinary tract infection, skin and soft tissue infection, and bacteremia (1). According to the report from the China Antimicrobial Resistance Surveillance System (http://www.carss.cn/Report/), the resistance of A. baumannii to carbapenem antibiotics has consistently increased in recent years. Additionally, in 2020, imipenem and meropenem resistance A. baumannii have reached 53.2% and 55.5%, respectively. Owing to its association with high mortality and lack of treatment options, carbapenem-resistant A. baumannii (CRAB) has been regarded by the World Health Organization as a critically important pathogen for the development of novel antibiotics (2–4).

The currently available treatment options for CRAB include minocycline, tigecycline, eravacycline, polymyxins, and cefiderocol (3, 5–7). The emergence of pandrug-resistant A. baumannii worldwide clearly demonstrates the impact of emerging resistance to last-resort antimicrobials (such as tigecycline and polymyxins) (8). Because of its strong bacteriostatic activity, tigecycline was considered the last line of defense against multidrug-resistant A. baumannii. Tigecycline acts as an inhibitor of protein synthesis by binding to 30S ribosomal subunits and blocking the insertion of tRNA into the A site of the ribosome during prokaryotic translation (9). However, the first case of tigecycline resistance was reported by Sader et al. in 2005 (10). Like polymyxins, chromosomal mechanisms of resistance in A. baumannii can lead to the rapid emergence of resistance during treatment with last-line antibiotics, including cefiderocol and tigecycline (11–13). The emergence of tigecycline-resistant A. baumannii has been reported worldwide and is increasing over time (14, 15). Therefore, more attention needs to be paid to the tigecycline resistance of A. baumannii. Additionally, its resistance mechanism needs to be studied to slow down the development of antibiotic resistance and provide clues for the research and development of new antibiotics.

So far, it has been reported that the mechanism of tigecycline resistance is mainly mediated by efflux pumps and regulatory factors. Tigecycline resistance is associated with resistance-nodulation-cell division (RND)-type transporters, mainly the AdeABC, AdeFGH, and AdeIJK efflux pumps, but other resistance mechanisms have also been implicated (16). Overexpression of AdeABC also confers resistance to carbapenems (17). In Gram-negative bacteria, overexpression of RND efflux pumps such as AdeABC, AdeFGH, AdeIJK, MexXY, and AcrAB is an important molecular mechanism in the resistance of bacteria to tigecycline (18). In addition, the mechanisms for the decreased sensitivity of tigecycline include the structural change in ribosomal protein S10 encoded by the *rpsJ* gene, which leads to a decreased affinity between tigecycline and the ribosome. Deletion of the *abrp* gene encoding a peptidase C13 family increases the permeability of the cell membrane to tigecycline. Mutations in SAM-dependent methyltransferase *trm* have also been found to decrease tigecycline susceptibility (19). The aforementioned mechanisms of drug resistance are mainly involved in chromosome-mediated tigecycline resistance (20). Recently, the plasmid-mediated tigecycline resistance gene *tet*(X3) was discovered in A. baumannii. The *tet*(X) gene has been shown to encode a flavin-dependent monooxygenase that can modify tigecycline. Tet(X3) can inactivate all tetracyclines and glycylcyclines, including tigecycline and the new FDA-approved elavacycline and omacycline. Analysis confirmed that *tet*(X3) is present in clinical bacteria, even in those that carry the *bla*_NDM-1_ gene, resulting in resistance to both tigecycline and carbapenems (6).

Although there have been many case reports and molecular typing epidemiological studies on tigecycline-resistant A. baumannii infection, little is known about the development of tigecycline resistance within the host in clinical isolates of A. baumannii. Hence, it is essential to investigate the evolutionary progress of A. baumannii within the host, providing evidence that can be used for the early diagnosis and treatment of patients infected with A. baumannii. Additionally, analyzing the influence of tigecycline on the genotype and phenotype of A. baumannii in the host (such as antibiotic resistance, adaptability, and virulence) will aid in finding new options for slowing down and preventing the development of multidrug resistance in A. baumannii.

## RESULTS

### Characteristics *of* A. baumannii isolates from patients with tigecycline treatment.

**FIG 1:**
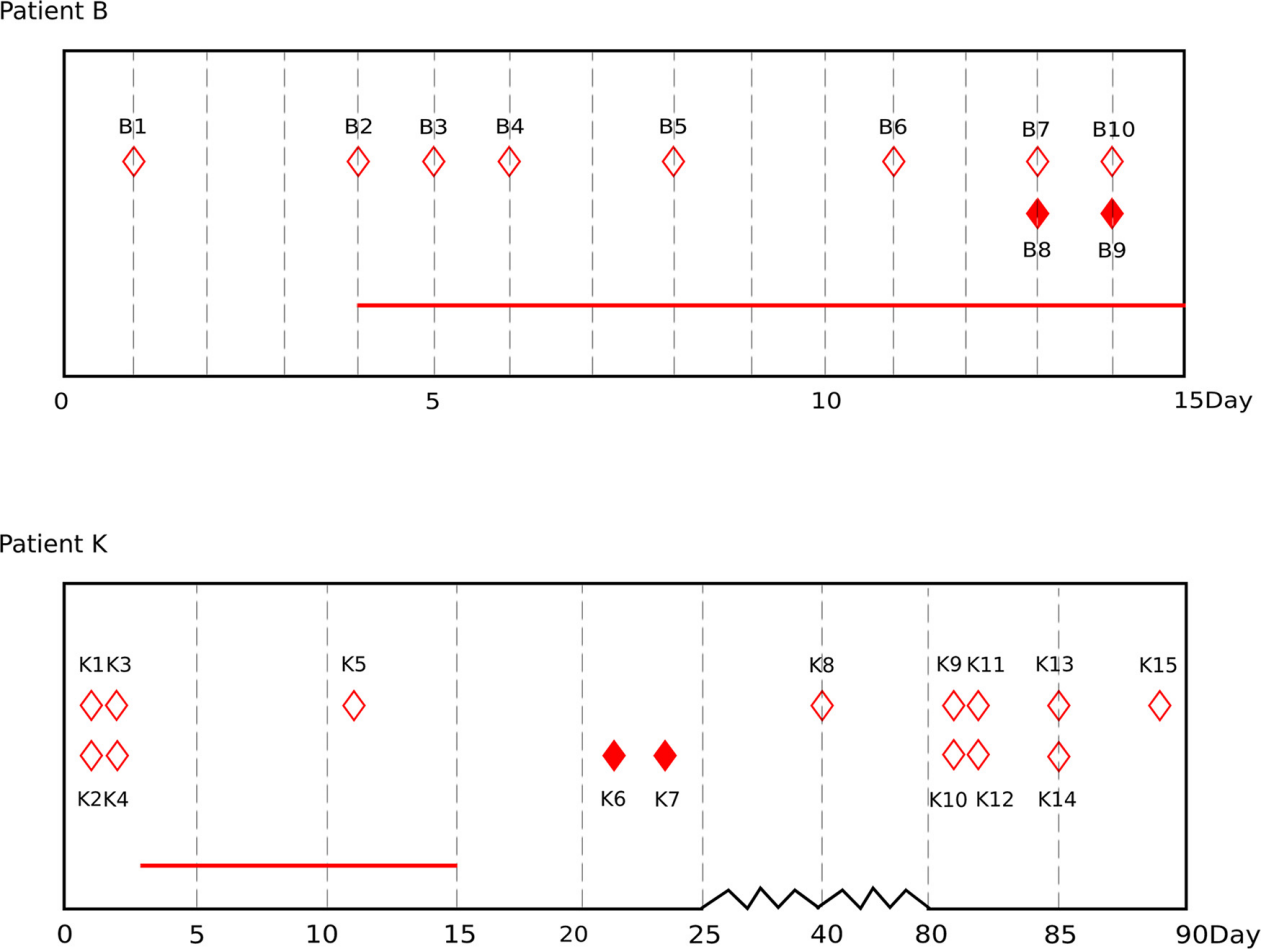
Clinical information of A. baumannii in two critically ill patients. The red solid diamonds indicated tigecycline-resistant isolates, and the red hollow diamonds indicated tigecycline-susceptible isolates. The sampling times were listed on the bottom, and the first isolate in each patient was set on day 1. The red horizontal line indicated the time period of tigecycline treatment.

Two patients (B and K) with A. baumannii infection treated with tigecycline were enrolled in this study. Patient B had severe pneumonia, and patient K had bacteremia. The two patients were admitted to the intensive care unit (ICU) and subsequently developed septic shock. Patient B died of severe pneumonia. Patient K was transferred to another hospital with an unknown prognosis. Patient B and patient K were administered tigecycline for 12 days and 13 days, respectively. Both patients received intravenous doses of tigecycline with 100-mg infusion followed by 50- mg infusions every 12 h. The tigecycline therapy data and patient characteristics are shown in Fig. 1 and Table S1.

A total of 10 isolates from patient B and 15 isolates from patient K were collected during hospitalization. The isolates of patient B were all collected from the respiratory tract. The isolates of patient K were isolated from blood or drainage. B7 and B8, B9 and B10, and K9 and K10 were isolated from the same sample, respectively. K1 and K2, K3 and K4, and K11 and K12 were isolated from blood samples obtained from different puncture sites on the same day. The genotypes and phenotypes of these isolates were analyzed. The common phenotype of A. baumannii was white, round, and smooth, with neat edges; however, some isolates showed a special phenotype. B8 and B9 showed a mucoid phenotype with wet colonies and unclear edges. K10 and K15 showed flat and off-white phenotypes (Fig. S1). All isolates were classified as CRAB and resistant to most antibiotics but were susceptible to colistin. Among them, B8, B9, K6, and K7 were resistant to tigecycline, and the other isolates were susceptible to tigecycline. The levofloxacin and minocycline MICs were 8 and 4 times higher in the tigecycline-resistant isolates isolated from patient K. The MICs of the other antibacterial drugs did not differ considerably within the same patient. Before the emergence of tigecycline-resistant isolates, patient B was also treated with meropenem (6 days) and linezolid (4 days), and patient K was treated with imipenem (4 days), meropenem (9 days), vancomycin (2 days), and polymyxin B (9 days). However, levofloxacin and minocycline were not used in the treatment of patient K. The tigecycline-resistant isolates were not isolated from patient K after withdrawal of tigecycline treatment. The results of the antimicrobial susceptibility tests are presented in Table S1.

### Phylogenetic and genotypic analyses of CRAB isolates.

**FIG 2:**
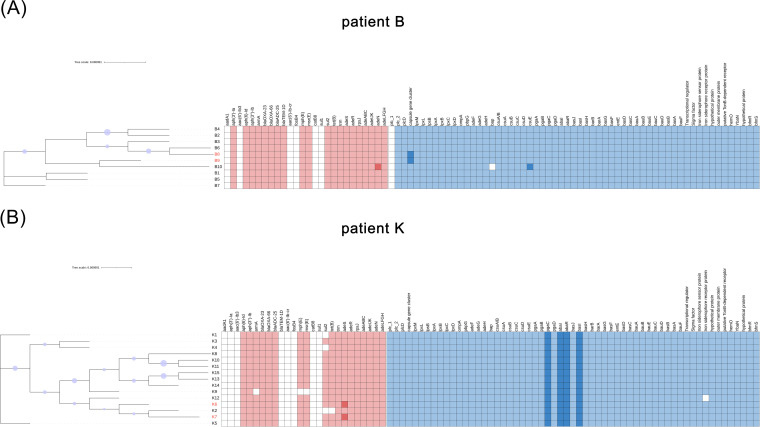
(A and B) Characteristics of antibiotic resistance and virulence genes in pre- and post-tigecycline therapy isolates of (A) patient B and (B) patient K. The phylogenetic tree was constructed based on core single-nucleotide variants. The red and blue colors indicated the presence of the resistance and virulence genes, and a different color indicated a different variant. The white color indicated the absence of the genes. The tigecycline-resistant isolates were highlighted in red.

Based on the multilocus sequence type (MLST) scheme of the Pasteur Institute, all 25 A. baumannii isolates from patients B and K were sequence type (ST) 2, which belonged to clonal complex CC2. According to the Oxford MLST scheme, A. baumannii isolates from patient B were all ST208, and those from patient K were all ST938, both of which belonged to clonal complex CC208. Phylogenetic tree analysis revealed that the isolates from the same patient were highly homologous (Fig. 2). The difference between tigecycline-resistant and -susceptible isolates was not evident.

The common antibiotic resistance genes and virulence-related genes of each A. baumannii isolate from patients B and K are shown in Fig. 2. The antibiotic resistance genes of each A. baumannii isolate from patient B were similar. As for patient K, K2 lacked *sul2* and *tet*(B), K9 lacked *armA*, *mph*(E), and *msr*(E), and K1 and K4 lacked *sul2*. Virulence-related genes were present in most isolates, but B10 lacked *bap*, and K12 lacked one iron siderophore receptor protein. There were variants of *adeN* and *csuE* in B10, variants of capsule gene cluster in tigecycline-resistant isolates B8 and B9, and *adeS* in tigecycline-resistant isolates K6 and K7, which were different from other isolates of the same patient.

### Changes in fitness.

**FIG 3:**
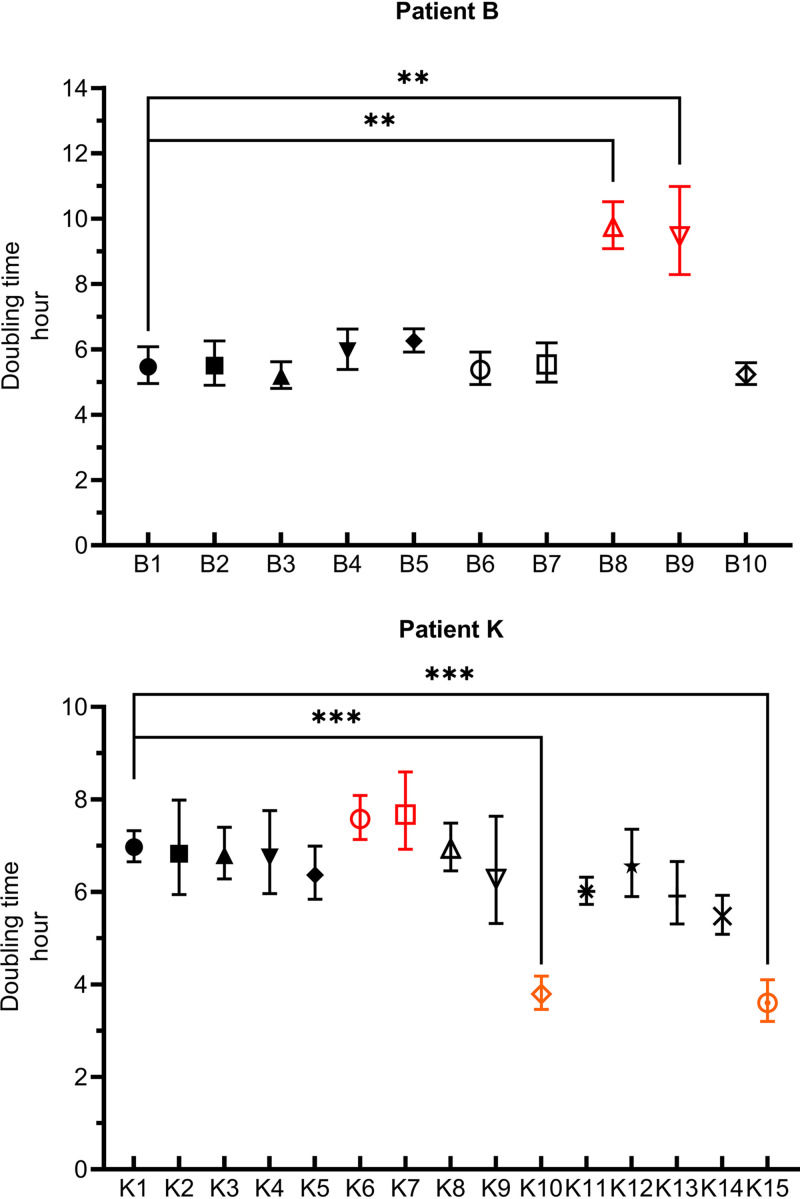
Doubling time of pre- and post-tigecycline therapy isolates in two patients. The tigecycline-resistant isolates were highlighted in red. The serum susceptible isolates were highlighted in orange. Experiments were performed in triplicate. Error bars represented the standard deviation. The asterisks indicated a significant difference (**, *P* < 0.05; ***, *P* < 0.01) compared with the time of the first isolate in each patient.

To observe the changes in the fitness of these isolates, we observed their growth *in vitro*. The doubling times of the bacteria are shown in Fig. 3. In patient B, the growth rates of tigecycline-resistant and mucoid isolates B8 and B9 were significantly lower than that of B1 (*P* < 0.05). In patient K, the growth rates of tigecycline-resistant isolates K6 and K7 were lower than that of K1, and the growth rates of flat isolates K10 and K15 were significantly higher than those of K1 (*P* < 0.01).

### *In vitro* and *in vivo* determination of virulence phenotype.

**FIG 4:**
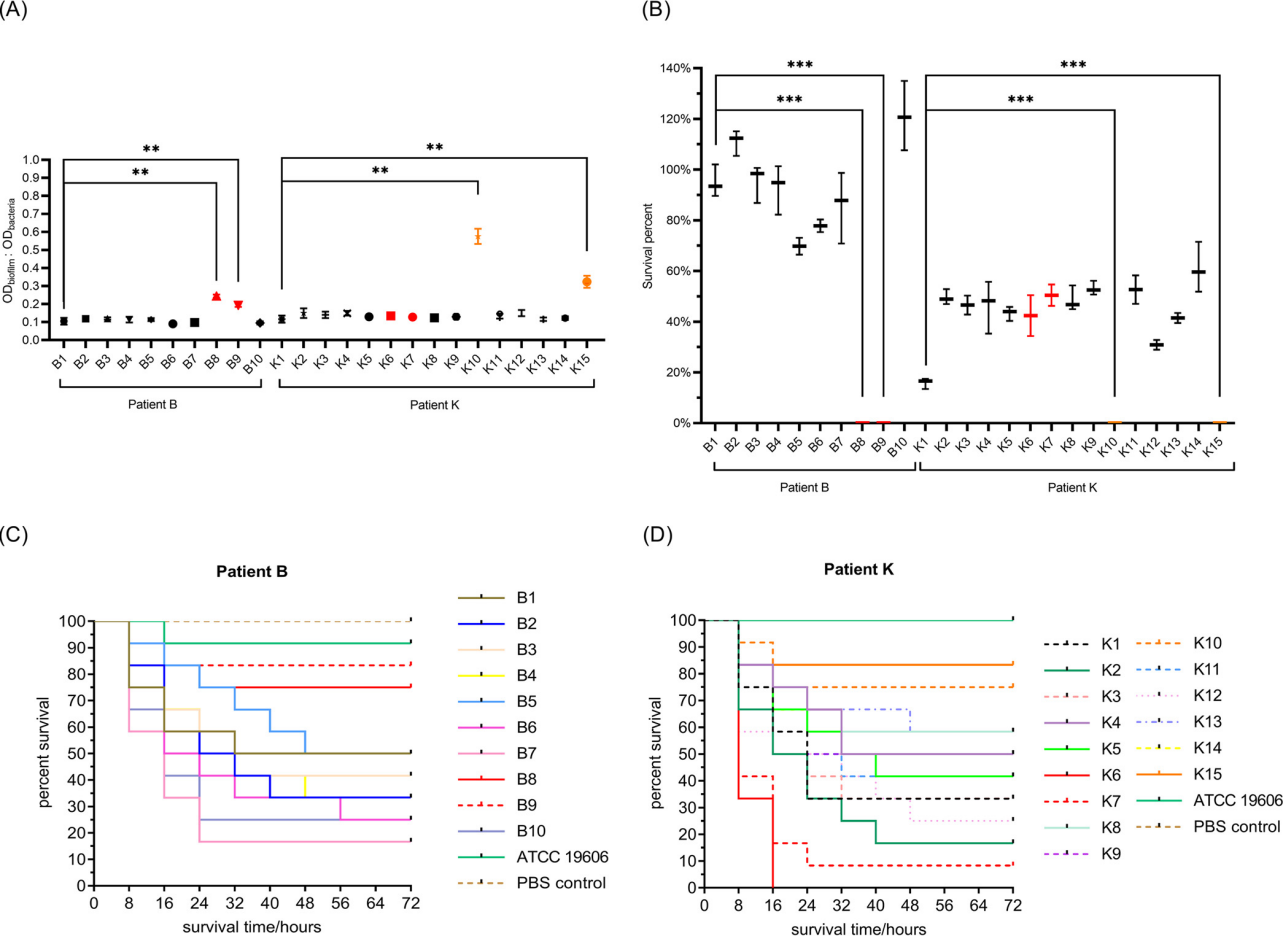
Virulence phenotype of pre- and post-tigecycline therapy isolates in two patients. (A) Biofilm formation assay; (B) serum bactericidal test. Experiments were performed in triplicate. Error bars represent the standard deviation. The asterisks indicated a significant difference (**, *P* < 0.05; ***, *P* < 0.01) compared with the time of the first isolate in each patient. (C and D) Survival of G. mellonella larvae (*n* = 20 per group) at a final concentration of 5 × 10^5^ cells/larva. The tigecycline-resistant isolates were highlighted in red. The serum susceptible isolates were highlighted in orange.

Biofilm formation ability in tigecycline-resistant and mucoid isolates B8 and B9 was stronger than that of B1 (*P* < 0.05); there was no statistical difference between the other seven isolates. The experimental results and statistical analyses are presented in Fig. 4A. Biofilm formation in flat isolates K10 and K15 was stronger than that in K1 (both *P* values < 0.05); there was no statistical difference between the other 12 isolates and K1.

The results of the serum bactericidal tests are shown in Fig. 4B. After incubation with serum for 1 h, the tigecycline-resistant and mucoid isolates, B8 and B9, were observed to be extremely susceptible to the bactericidal effect of serum. However, this phenotype was not observed in the tigecycline-resistant isolates of patient K. The flat isolates, K10 and K15, were completely inhibited in the serum.

The survival results of Galleria mellonella larvae are shown in Fig. 4C and D. ATCC 19606, which has weak virulence, was used as a negative control. The phosphate-buffered saline (PBS)-injected group was used as the blank group. The log-rank test results showed that there was no difference in survival rate between the larvae infected with B8, B9, K10, or K15 and the larvae infected with ATCC 19606 (all *P* > 0.05), indicating that the virulence of these four isolates was relatively weak. However, the survival rate differed between the larvae infected with the other eight tigecycline-susceptible isolates in patient B and the larvae infected with ATCC 19606, suggesting that the virulence of the eight tigecycline-susceptible isolates in patient B was stronger. As for patient K, the virulence of the two tigecycline-resistant isolates, K6 and K7, was notably stronger than that of the tigecycline-susceptible isolates.

### Genomic comparative analysis of CRAB within the host.

Among the two patients, 23 single nucleotide variations (SNVs) were identified (Tables S2 and S3). A total of six mutations were observed in patient B, three nonsynonymous SNVs, one frameshift mutation, one synonymous SNV, and one intergenic SNV. A total of 17 SNVs were observed in patient K, 6 nonsynonymous SNVs, 6 intergenic SNV, 4 frameshift mutations, and 1 stop-gain mutation.

Tigecycline resistance-related mutations were identified in *baeR*, *wzc*, *aroQ*, *rluC*, and *adeS*. In patient B, three SNVs were present in the tigecycline-resistant isolates B8 and/or B9. The insertion of 27 bp in B8 resulted in a frameshift mutation in *baeR*. Both B8 and B9 harbored a C-to-T point mutation in the upstream noncoding region of *aroQ* and amino acid substitution of leucine for proline at amino acid 541 in *wzc*, which is related to the biosynthesis of a bacterial capsular polysaccharide. In patient K, a nonsynonymous SNV of *adeS* (Ala94Thr) and an upstream mutation of *rluC* were detected in two tigecycline-resistant isolates. Serum susceptible-related mutations were identified in *wzc*, *aroQ*, and *itrA2*. Both *wzc* and *itrA2* are bacterial capsular polysaccharide-related genes. The mutations in *wzc* and *aroQ* can be described as tigecycline resistance-related mutations. The *itrA2* encoding gene had a missense mutation of C to T, resulting in the substitution of glycine with glutamic acid at amino acid 188. Gene function annotation results suggested that tigecycline resistance and virulence-related genes were related to transcription, signal transduction mechanisms, amino acid transport and metabolism, translation, ribosomal structure and biogenesis, and cell wall/membrane/envelope biogenesis. The tigecycline resistance-related mutations were not detected in tigecycline-susceptible isolates. The serum susceptible-related mutations were also not detected in normal phenotype isolates. The results are presented in Table 1.

**TABLE 1:** SNVs associated with tigecycline resistance and virulence phenotypes in two patients exposed to tigecycline

Phenotype	Patient (isolate)	Gene	Annotation	COG category^*a*^	SNV type	Protein change^*b*^
Tigecycline resistance	B (B8)	*baeR*	Response regulator BeaR	K	Frameshift mutation	Nine amino acids inserted at position 113
	B (B8 B9)	*wzc*	Tyrosine protein kinase	D	Missense mutation	Pro541Leu
	B (B8 B9)	*aroQ*	Type II 3-dehydroquinate dehydratase	E	Upstream mutation	
	K (K6 K7)	*adeS*	Two-component sensor histidine kinase AdeS	T	Missense mutation	Ala94Thr
	K (K6 K7)	*rluC*	23S rRNA pseudouridine synthase RluC	J	Upstream mutation	
	K (K6 K7)	IS*Aba1*	Transposase	L	Acquisition	
Serum susceptible	B (B8 B9)	*wzc*	Tyrosine protein kinase	D	Missense mutation	Pro541Leu
	B (B8 B9)	*aroQ*	Type II 3-dehydroquinate dehydratase	E	Upstream mutation	
	K (K15)	*itrA2*	Bacterial sugar transferase	M	Missense mutation	Gly188Glu

a COG categories: K, transcription; D, cell cycle control, cell division, chromosome partitioning; T, signal transduction mechanisms; E, amino acid transport and metabolism; J, translation, ribosomal structure, and biogenesis; L, replication, recombination, and repair; M, cell wall/membrane/envelope biogenesis.

b SNVs of the genomes were identified by mapping sequence reads for each isolate against the first isolate in each patient.

**FIG 5:**
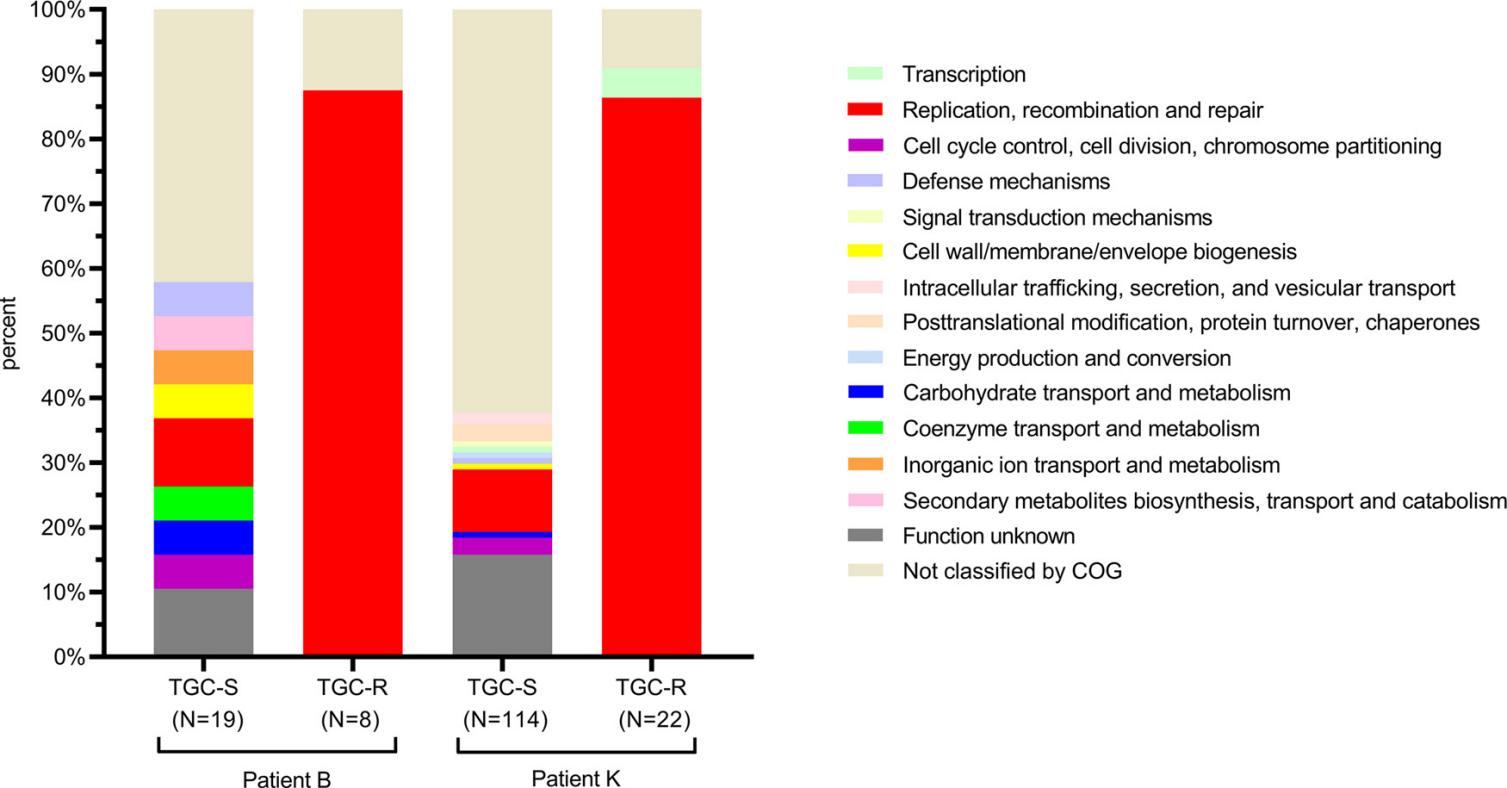
COG functional category enrichment among differential core genes of tigecycline-resistant and tigecycline-susceptible isolates. Distributions in each category were compared with all genes in each group. N indicated the number of differential core genes.

Gene content analysis revealed that 91.2% (3,632/3,983) and 90.6% (3,553/3,922) of the genes or gene clusters were observed in all isolates in patients B and K (Table S4). In patients B and K, 0.48% (19) to 2.91% (114) of the genes or gene clusters were present in all tigecycline-susceptible isolates but absent in one of two tigecycline-resistant isolates from each patient, which were classified as TGC-S (Fig. 5 and Table S4). However, no specific genes or gene clusters were detected in all tigecycline-susceptible isolates and absent in all tigecycline-resistant isolates. Meanwhile, 0.20% (8) and 0.56% (22) of the genes or gene clusters were present in all tigecycline-resistant isolates but were absent in more than half of the tigecycline-susceptible isolates obtained from each patient. These genes or gene clusters were classified as TGC-R (Fig. 5 and Table S4). Only one specific gene cluster, which was annotated as *IS*Aba1, was identified in the tigecycline-resistant isolates and absent in all tigecycline-susceptible isolates from patient K (Table 1). Clusters of Orthologous Group (COG) functional categories were assigned to each of these genes or gene clusters, and then the COG category distribution was compared (Fig. 5). TGC-S groups showed more types of classification than TGC-R. Genes or gene clusters in TGC-R were enriched for “replication, recombination and repair” categories. The altered genes in these categories mainly were transposase.

## DISCUSSION

To observe and analyze the progression of tigecycline resistance within the host, we collected multiple A. baumannii isolates from two ICU patients with A. baumannii infection who underwent tigecycline therapy.

The mechanism of tigecycline resistance *in vivo* primarily involves the upregulation of adeABC efflux pumps, caused by the mutations in the regulatory genes *adeS* (mutational hot spots near histidine 149) and *adeR* (mutational hot spots in the DNA binding domain) (20). Other mechanisms that do not involve the adeABC pumps were also proposed in some cases through a combined genomic and transcriptomic approach (21, 22). In our study, we observed genomic variations in *baeR*, *wzc*, *aroQ*, *rluC*, and *adeS*. The emergence of tigecycline resistance was associated with the disruption of AdeS by *IS*Aba1 or *adeS* mutations (Ala94Val, Ser8Arg), which resulted in the overexpression of adeABC efflux pumps (23–25). A novel tigecycline resistance-related *adeS* mutation, Ala94Thr, was first identified in our study. A previous study identified that the two-component regulatory system, BaeSR, influences the tigecycline susceptibility of A. baumannii through the positive regulation of the resistance-nodulation-division efflux pump genes *adeA* and *adeB*. The overexpression of *baeR* resulted in a doubled tigecycline MIC, with a more than 2-fold increase in *adeA* and *adeB* expression (26). In our study, nine amino acids inserted in BaeR were identified among tigecycline-resistant isolates, which may cause protein function inactivation or expression changes. The *rluC* gene of Escherichia coli encodes a pseudouridine synthase that is solely responsible for the synthesis of pseudouridine at positions 955, 2504, and 2580 in 23S rRNA (27). Further studies are required to clarify the role of *rluC* in tigecycline resistance in A. baumannii. *AroQ* and *wzc* were associated with tigecycline resistance and virulence in our study. The *aroQ* gene encodes a periplasmic chorismate mutase. Chorismate mutases are generally involved in the synthesis of tyrosine and phenylalanine and are key to the synthesis of a plethora of secondary metabolites. The PmrAB two-component regulatory system is required for Salmonella enterica serovar Typhimurium virulence. PmrAB-controlled modifications of the lipopolysaccharide layer confer resistance to cationic antibiotic polypeptides, which may allow bacteria to survive within macrophages. An *in silico* analysis identified *aroQ* as a PmrAB target gene in *S.* Typhimurium, suggesting that the unknown function of AroQ might be involved in bacterial-host interactions (28). The comparison of antibiotic resistance and virulence between matt and mucoid colonies of Klebsiella pneumoniae coproducing NDM-1 and OXA-232 isolated from a single patient indicated the insertion of IS*5* in the *wzb* gene of two matt-type isolates. The matt-type isolates were resistant to tigecycline. *In vivo* and *in vitro* virulence assays indicated that the mucoid-type isolates were significantly more virulent than the matt-type isolates (29). Previous reports have suggested that, in A. baumannii, *wzc* is critical for the assembly of capsule polysaccharides, indicating a possible mechanism of antibiotic resistance (30). Wza affects the expression of other proteins of the Wzy capsule polysaccharide synthesis pathway, which affects the assembly, export, and extracellular fixation of capsular polysaccharide, resulting in synergistic effects that decrease bacterial virulence (31). Our study suggests that tigecycline resistance in A. baumannii may be related to multiple proteins, which provides a potential exploration target for specific research on the antibiotic resistance of tigecycline. Another capsule polysaccharide gene, *itrA2*, is involved in the virulence phenotype. A recent study indicated that capsular structure could determine virulence among A. baumannii isolates by altering bacterial interactions with host complement-mediated opsonophagocytosis (32).

Few studies have examined the phenotypic changes in A. baumannii after tigecycline treatment. In our study, the emergence of tigecycline resistance *in vivo* was associated with the increased MICs of levofloxacin and minocycline in one patient. After the withdrawal of tigecycline pressure, the tigecycline-resistant isolates were not isolated from patient K. We hypothesized that tigecycline resistance in patient K is a transient phenotype, which is in agreement with the findings of another study (33). Previous studies have revealed different effects of emergent tigecycline resistance on virulence based on the G. mellonella model. In the two cases, tigecycline-resistant isolates exhibited increased virulence in one patient; however, in another patient, the opposite results were identified (25). In our study, tigecycline-resistant A. baumannii emerged after tigecycline treatment, which had a lower cost of fitness compared with the tigecycline-susceptible isolates in each patient. The *in vivo* virulence of the isolates showed diametrically opposite results in two patients, which is similar to a previous study (25). After tigecycline treatment, isolates with a distinct phenotype emerged. The mucoid phenotype was accompanied by the appearance of resistance to tigecycline; however, the flat phenotype was not related to tigecycline resistance. The mucoid and flat isolates exhibited decreased virulence *in vivo* and were more susceptible to serum but exhibited increased biofilm formation ability. Our research provides a theoretical basis for elucidating the formation of tigecycline resistance in multidrug-resistant A. baumannii.

This retrospective study had some limitations. The sample collection interval varied among the two patients, which may have resulted in the loss of some evolution information. A larger data set and more comprehensive screening are required to determine whether the findings described here can be more generalized. The functional verification of tigecycline resistance and virulence-related genes has not been studied in depth. Most variant genes involved transcription, translation, signal transduction mechanisms, amino acid transport, and metabolism-related genes, which indicated potential transcription or translation regulation in tigecycline resistance and virulence evolution of A. baumannii. Transcriptome and proteome research should be carried out in future studies, as regulatory networks and signal transduction proteins are important for the survival of A. baumannii within the host (34–36).

**Conclusions.** In our study, the development of tigecycline resistance *in vivo* was accompanied by an increase in the fitness cost. The virulence changes in different patients were reasonably different, suggesting that the host may play a role in tigecycline resistance and virulence evolution. Several efflux pump and capsular polysaccharide-related genes are associated with tigecycline resistance and the evolution of virulence in A. baumannii within the host. Further studies are required for the evaluation of the functionality of these genes.

## MATERIALS AND METHODS

### Clinical A. baumannii isolates.

From November 2018 to April 2019, a total of 25 sequential A. baumannii isolates were collected from two patients in the Department of Intensive Care Unit (ICU), Peking University People’s Hospital. These isolates were cultured from specimens obtained from sputum, blood, and drainage fluid.

### *In vitro* bacterial identification and antimicrobial susceptibility tests.

A. baumannii isolates were identified using a Vitek 2 compact biochemical identification system (bioMérieux, France) and matrix-assisted laser desorption–ionization time of flight mass spectrometry (MALDI-TOF MS) (Bruker Daltonics, Germany). Antimicrobial susceptibility was determined by the agar dilution method and broth microdilution method (for tigecycline and colistin), and the results (excluding tigecycline) were interpreted according to the MIC interpretive breakpoints recommended by the Clinical and Laboratory Standards Institute (CLSI) (37). The tigecycline susceptibility results were interpreted following the guidelines of the U.S. Food and Drug Administration for *Enterobacteriaceae* (susceptible [S], ≤2 mg/L; intermediate [I], 4 mg/L; resistant [R], ≥8 mg/L). The tested antimicrobial agents included imipenem, meropenem, amikacin, levofloxacin, ciprofloxacin, ceftriaxone, ceftazidime, piperacillin-tazobactam, minocycline, tigecycline, and colistin. The reference isolates Escherichia coli ATCC 25922 and Pseudomonas aeruginosa ATCC 27853 were used as quality control isolates.

### *In vitro* biofilm formation assay.

Biofilm formation was determined by incubating an overnight culture (diluted 1:100 in fresh LB broth) in 96-well plates, at 37°C for 24 h, without shaking (38). Biofilms were stained with 1% (wt/vol) crystal violet, solubilized with 95% ethanol, and then quantified at 570 nm and recorded as the optical density (OD_biofilm_). At the same time, the OD value at 600 nm of the bacterial suspension was also measured and recorded as OD_bacteria_. Then the ratio of OD_biofilm_ to OD_bacteria_ was calculated. Each group had four replicates. The experiments were performed in triplicate.

### Growth curve analysis.

Fitness was investigated using a growth curve assay, as previously described (39). All the isolates were cultured overnight in LB broth, diluted to an OD at 600 nm (OD_600_) of 0.01, and incubated at 37°C with vigorous shaking (200 rpm). Cell density was measured hourly at 600 nm. The doubling time was calculated according to the data from the exponential growth phase using Prism software (GraphPad Software, Inc., USA). Each group had three replicates. The experiments were performed in triplicate.

### Serum bacterial test.

A serum killing assay was conducted to determine virulence *in vitro*, as previously described (40). An inoculum of 25 μL was prepared from a mid-log-phase culture and added to 75 μL of pooled human sera in a polypropylene tube. Viable counts were checked at 0 h and 1 h after incubation at 37°C. Each group had three replicates. The experiments were performed in triplicate.

### Galleria mellonella lethality assay.

The virulence of A. baumannii isolates was assessed *in vivo* using G. mellonella as an insect model for experimental infection. Bacteria cultured in LB broth were collected by centrifugation and suspended in phosphate-buffered saline (PBS). The number of viable cells in each inoculum was determined as CFU on LB agar plates. G. mellonella larvae weighing 250 to 350 mg were randomly selected and grouped for killing assays with 20 larvae per group. The larvae were injected with 10 μL of bacterial suspension (5 × 10^7^ CFU/mL) into the hemocoel via the penultimate left proleg of each larva. Twenty larvae in the negative-control group were injected with the same volume of PBS. All larva groups were incubated at 37°C in the dark and monitored every 4 h for a total of 3 days. Larvae were considered dead when they did not respond to needling (41).

### Whole-genome sequencing and bioinformatics analysis.

All A. baumannii isolates were sequenced using Illumina technology with 150-bp paired-end protocols on a NextSeq 500 system. Two isolates, B10 and K7, were sequenced using an RS II DNA sequencing system (Pacific Biosciences, Menlo Park, CA, USA). *De novo* assembly of the genomes of all isolates in this study was performed using Velvet (Ridom GmbH, Münster, Germany) (42) and annotated using Prokka software (43) and Rapid Annotation using Subsystems Technology (RAST; https://rast.nmpdr.org/) (44). Single-nucleotide variant analysis was performed and filtered using Genome Analysis Toolkit software with the default mapping parameters (45). For phylogenetic analysis, the core genome was extracted using Roary software (46). After recombination region removal, RAxML was used to build a phylogenetic tree (47). The COG functional categories were identified using the eggNOG-Mapper with the default parameters (http://eggnog-mapper.embl.de/) (48). All annotations were visualized using iTOL (https://itol.embl.de/) (49).

### Statistical analysis.

Statistical analysis was performed using Prism software (GraphPad Software, Inc., USA). Biofilm-forming ability was compared using the Mann-Whitney test. Comparison of serum bactericidal activity and doubling time was assessed using an unpaired *t test*. Comparison of survival curves between Galleria mellonella larvae was determined using the log rank test. Statistical significance was set at *P < *0.05, and all tests were two-tailed.

### Ethics statement.

The study was reviewed and approved by the Ethical Review Committee of Peking University People’s Hospital (number 2018PHB187). Informed consent was waived, because the medical records and patient information were anonymously reviewed and collected in this observational study.

### Data availability.

This whole-genome shotgun project has been deposited at GenBank under the accession numbers JAHWVM000000000 (B1), JAHWVL000000000 (B2), JAHWVK000000000 (B3), JAHWVJ000000000 (B4), JAHWVI000000000 (B5), JAHWVH000000000 (B6), JAHWVG000000000 (B7), JAHWVF000000000 (B8), JAHWVE000000000 (B9), CP079942 (B10), JAHWWA000000000 (K1), JAHWVZ000000000 (K2), JAHWVY000000000 (K3), JAHWVX000000000 (K4), JAHWVW000000000 (K5), JAHWVV000000000 (K6), CP079945 (K7), JAHWVU000000000 (K8), JAHWVT000000000 (K9), JAHWVS000000000 (K10), JAHWVR000000000 (K11), JAHWVQ000000000 (K12), JAHWVP000000000 (K13), JAHWVO000000000 (K14), and JAHWVN000000000 (K15).
