# Supplementary material for: Genomic and Phenotypic Evolution of Tigecycline-Resistant Acinetobacter baumannii in Critically Ill Patients
Source: Microbiol Spectr. 2022 Jan 19;10(1):e01593-21. doi: 10.1128/spectrum.01593-21 (PMC8768575; doi:10.1128/spectrum.01593-21)
Supplement: SUPPLEMENTAL FILE 1 — Supplemental material. Download Spectrum01593-21_Supplementary_Materials_update.pdf, PDF file, 0.3 MB [file spectrum01593-21_supplementary_materials_update.pdf]

Supplementary Materials

Figure S1. Colony morphology of normal(A), mucoid (B) and flat (C) phenotype.

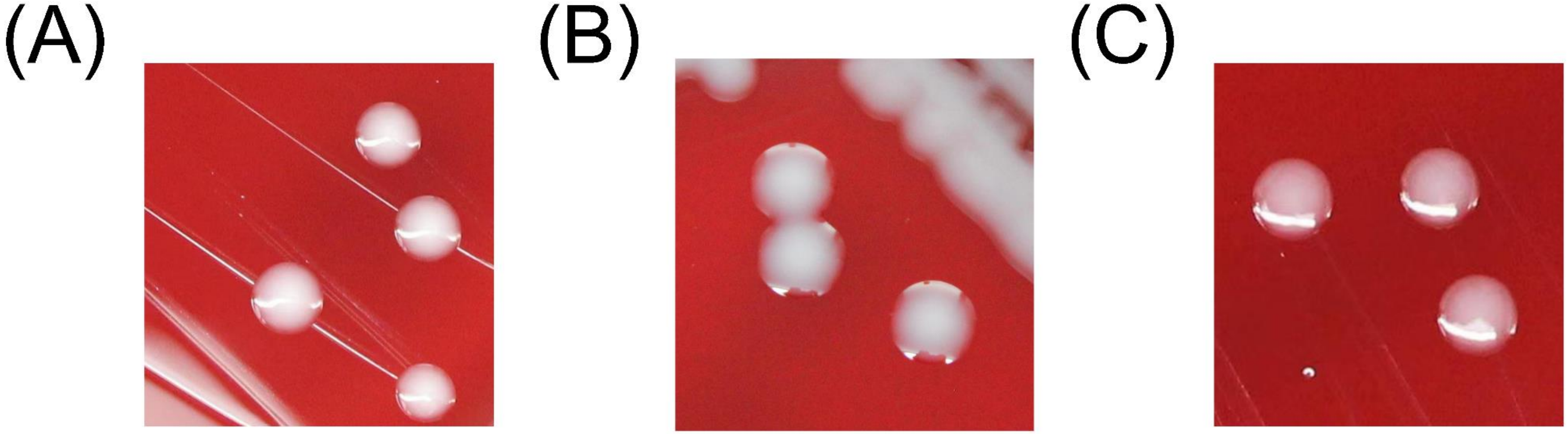

Table S1. Characteristics of isolates from two critically ill patients.

Patient	Isolate	Origin	Sampling time	Phenotype	MIC (mg/L)										
					Meropenem	Imipenem	Ceftazidime	Cefatriaxone	Piperacillin-Tazobactam	Ciprofloxacin	Levofloxacin	Amikacin	Minocycline	Tigecycline	Colistin
B	B1	Bronchoalveolar lavage fluid	2018/11/30	normal	>32	>32	>256	>256	>256	64	8	>256	8	1	0.25
	B2	Sputum	2018/12/3	normal	>32	>32	>256	>256	>256	32	8	>256	8	1	0.25
	B3	Sputum	2018/12/4	normal	>32	>32	>256	>256	>256	64	8	>256	8	1	0.25

	B4	Sputum	2018/12/5	normal	>32	>32	>256	>256	>256	>64	8	>256	8	1	0.25
	B5	Sputum	2018/12/7	normal	>32	>32	>256	>256	>256	64	8	>256	8	1	0.25
	B6	Sputum	2018/12/10	normal	>32	>32	>256	>256	>256	64	8	>256	8	2	0.25
	B7	Sputum	2018/12/12	normal	>32	>32	>256	>256	>256	64	8	>256	8	2	0.25
	B8	Sputum	2018/12/12	mucoid	>32	>32	>256	>256	>256	64	8	>256	8	8	0.25
	B9	Sputum	2018/12/13	mucoid	>32	>32	>256	>256	>256	64	8	>256	8	8	0.25
	B10	Sputum	2018/12/13	normal	>32	>32	>256	>256	>256	>64	16	>256	16	2	0.25
K	K1	Blood	2019/1/31	normal	32	32	>256	>256	>256	64	8	128	8	2	0.25
	K2	Blood	2019/1/31	normal	32	32	>256	>256	>256	64	8	128	8	2	0.25
	K3	Blood	2019/2/1	normal	>32	>32	>256	>256	>256	64	8	128	8	2	0.25
	K4	Blood	2019/2/1	normal	32	32	>256	>256	>256	64	8	128	8	2	0.25
	K5	Blood	2019/2/10	normal	>32	>32	>256	>256	>256	64	8	256	8	2	0.25
	K6	Drainage	2019/2/20	normal	32	>32	>256	>256	>256	>64	64	128	32	>8	0.25

K7	Blood	2019/2/22	normal	32	32	>256	>256	>256	>64	64	128	32	>8	0.25
K8	Drainage	2019/3/11	normal	>32	>32	>256	>256	>256	64	8	128	8	2	0.25
K9	Drainage	2019/4/21	normal	>32	>32	>256	>256	>256	64	8	128	8	2	0.25
K10	Drainage	2019/4/21	flat	>32	>32	>256	>256	>256	64	8	128	8	2	0.25
K11	Blood	2019/4/22	normal	>32	>32	>256	>256	>256	64	8	256	8	2	0.25
K12	Blood	2019/4/22	normal	>32	>32	>256	>256	>256	64	16	128	8	2	0.25
K13	Blood	2019/4/21	normal	>32	>32	>256	>256	>256	64	8	256	8	2	0.25
K14	Blood	2019/4/25	normal	>32	>32	>256	>256	>256	64	8	256	8	2	0.25
K15	Blood	2019/4/29	flat	>32	>32	>256	>256	>256	64	8	256	8	2	0.25

Table S2. Whole genome sequencing metadata.

Isolate ID	WGS technology	Genome size (bp)	Total no. of reads (M)	No. of contigs	N_{50} length	Avg reference coverage	SRA_bioproject	SRA_biosample	GenBank accession number
B1	Illumina	4,046,717	6.940940	67	116,652	137	PRJNA747802	SAMN20295781	JAHWVM000000000
B2	Illumina	4,047,749	7.273966	67	116,652	144	PRJNA747802	SAMN20295782	JAHWVL000000000
B3	Illumina	4,027,461	7.924860	67	116,652	156	PRJNA747802	SAMN20295783	JAHWVK000000000
B4	Illumina	4,046,823	6.872576	67	128,295	137	PRJNA747802	SAMN20295784	JAHWVJ000000000
B5	Illumina	4,023,259	7.065968	69	116,505	137	PRJNA747802	SAMN20295785	JAHWVI000000000
B6	Illumina	4,047,240	8.358688	69	128,295	166	PRJNA747802	SAMN20295786	JAHWVH000000000
B7	Illumina	4,019,946	8.195314	66	127,221	160	PRJNA747802	SAMN20295787	JAHWVG000000000
B8	Illumina	4,024256	7.811020	60	128,295	154	PRJNA747802	SAMN20295788	JAHWVF000000000
B9	Illumina	4,046,396	7.163166	66	128,295	142	PRJNA747802	SAMN20295789	JAHWVE000000000

B10	Pacbio + Illumina	4,019,005	0.598110	2	4,010,274	142	PRJNA747802	SAMN20295790	CP079942
K1	Illumina	4,002,351	8.753508	55	132,027	165	PRJNA747803	SAMN20295811	JAHWWA000000000
K2	Illumina	4,002,097	8.753508	55	123,465	151	PRJNA747803	SAMN20295812	JAHWVZ000000000
K3	Illumina	4,014,494	6.614782	60	132,027	163	PRJNA747803	SAMN20295813	JAHWVY000000000
K4	Illumina	4,023,359	6.614782	52	133,166	118	PRJNA747803	SAMN20295814	JAHWVX000000000
K5	Illumina	4,036,380	8.753508	61	133,166	163	PRJNA747803	SAMN20295815	JAHWVW000000000
K6	Illumina	4,038,777	6.614782	57	133,166	107	PRJNA747803	SAMN20295816	JAHWVV000000000
K7	Pacbio + Illumina	4,020,619	0.929860	3	3,941,055	190	PRJNA747803	SAMN20295817	CP079945
K8	Illumina	4,011,590	6.614782	62	121,445	189	PRJNA747803	SAMN20295818	JAHWVU000000000
K9	Illumina	4,019,728	8.753508	62	122,946	149	PRJNA747803	SAMN20295819	JAHWVT000000000
K10	Illumina	4,020,699	8.753508	67	116,251	167	PRJNA747803	SAMN20295820	JAHWVS000000000

K11	Illumina	4,018,265	8.753508	62	121,807	118	PRJNA747803	SAMN20295821	JAHWVR000000000
K12	Illumina	4,009,506	8.753508	60	123,465	142	PRJNA747803	SAMN20295822	JAHWVQ000000000
K13	Illumina	4,014,191	6.614782	60	132,005	134	PRJNA747803	SAMN20295823	JAHWVP000000000
K14	Illumina	4,007,319	8.753508	60	131,688	185	PRJNA747803	SAMN20295824	JAHWVO000000000
K15	Illumina	4,018,114	6.614782	62	132,005	149	PRJNA747803	SAMN20295825	JAHWVN000000000

Table S3. Summary of SNVs.

Reference isolate	Position	SNV	Isolates with SNV
B10	122641	G → A	B1-B9
	653577	T → TTGTGCCTGAGCAGAAGCAAGATTAGCC	B8
	1615794	C → T	B8, B9
	1648279	T → C	B1-B9
	3903593	G → A	B2
	3939447	C → T	B8, B9
K7	193605	T → A	K12
	576762	G → T	K9
	681493	GT → G	K13, K14
	818850	A → C	K11
	1179956	G → A	K8-K11, K13-K15

1498877	T → TTGAACC	K4
1794650	G → A	K8-K11, K13-K15
1889205	T → C	K1-K5, K8-K15
2841936	AG → A	K12
3222090	AC → A	K15
3222100	A → T	K15
3222102	TG → T	K15
3222104	G → T	K15
3222107	G → GAGATT	K15
3459665	C → A	K1-K5, K8-K15
3722058	TA → T	K12
3852845	C → T	K15
